# Maintaining Sufficient Nanos Is a Critical Function for *Polar Granule Component* in the Specification of Primordial Germ Cells

**DOI:** 10.1534/g3.112.004192

**Published:** 2012-11-01

**Authors:** Girish Deshpande, Emma Spady, Joe Goodhouse, Paul Schedl

**Affiliations:** Department of Molecular Biology, Princeton University, Princeton, New Jersey 08544

## Abstract

Primordial germ cells (PGC) are the precursors of germline stem cells. In *Drosophila*, PGC specification is thought to require transcriptional quiescence and three genes, *polar granule component* (*pgc*), *nanos* (*nos*), and *germ cell less* (*gcl*) function to downregulate Pol II transcription. While it is not understood how *nos* or *gcl* represses transcription, *pgc* does so by inhibiting the transcription elongation factor b (P-TEFb), which is responsible for phosphorylating Ser2 residues in the heptad repeat of the C-terminal domain (CTD) of the largest Pol II subunit. In the studies reported here, we demonstrate that *nos* are a critical regulatory target of *pgc*. We show that a substantial fraction of the PGCs in *pgc* embryos have greatly reduced levels of Nos protein and exhibit phenotypes characteristic of *nos* PGCs. Lastly, restoring germ cell–specific expression of Nos is sufficient to ameliorate the *pgc* phenotype.

The germline of *Drosophila* arises from a special group of primordial germ cells (PGC). PGCs are formed during nuclear cycles 9 and 10 when nuclei migrate from the center of the embryo into the posterior pole plasm. These nuclei induce cellularization, incorporating the maternal germline determinants that are assembled in the pole plasm during oogenesis. In addition to precocious cellularization, PGCs differ from the surrounding soma in a number of important respects [reviewed in Santos and Lehmann (2004), Seydoux and Bruan (2006), and Wylie (1999)]. One of these is transcription. Whereas somatic nuclei turn on transcription, it is shut down in PGCs, and they remain transcriptionally quiescent until after they exit the gut much later in development (Zalokar 1976; Seydoux and Dunn 1997). Global downregulation of transcription in PGCs correlates with the phosphorylation status of the heptad repeats in the C-terminal domain (CTD) of the largest Pol II subunit. There are two Serine (Ser) residues in each heptad, Ser2 and Ser5, which are phosphorylated at different steps. Ser5 phosphorylation is coordinated with initiation, and Ser2 phosphorylation accompanies elongation (Phatnani and Greenleaf 2006; Hirose and Ohkuma 2007). While both of these modifications are elevated in somatic nuclei of blastoderm embryos, this is not true in PGCs: the elongation phosphorylation, PSer2, is absent, and there are only low levels of the initiation phosphorylation, PSer5 (Seydoux and Dunn 1997; Deshpande *et al.* 2005). Flies are not the only organism in which primordial germ cells downregulate transcription. Transcriptional quiescence is also a hallmark of germline progenitors in *C. elegans* and *Xenopus* (Seydoux and Dunn 1997; Lai *et al.* 2012).

The establishment of transcriptional quiescence in fly PGCs is mediated by at least three maternally deposited pole plasm determinants, *germ cell-less* (*gcl*), *polar granule component* (*pgc*), and *nanos (nos*) (Asaoka *et al.* 1998, 1999; Deshpande *et al.* 1999, 2004; Leatherman *et al.* 2002; Martinho *et al.* 2004). These three maternal factors have different (though potentially overlapping) gene targets for downregulation, act at slightly different times, and use different mechanisms. *gcl* functions when PGCs are formed and targets genes that are activated prior to the mid-blastula transition. *pgc* and *nos* are required later, and while they both prevent transcription of somatic mid-blastula transition genes, there are differences in their targets. *pgc* blocks *zen* and *tailess*, whereas *nos* is required to prevent pair rule genes like *even-skipped* from being activated. Blocking Pol II activity appears to be important for PGC development. While PGCs from *gcl* and *pgc* mothers can go on to form functional germline stem cells (GSC), the number of PGCs in coalesced stage 14–15 mutant gonads is substantially reduced. Even more drastic effects are evident in *nos* embryos. *nos* PGCs fail to maintain PGC identity, and unlike either *gcl* or *pgc* PGCs, they never develop into functional GSCs (Kobayashi *et al.* 1996; Sato *et al.* 2007). Further supporting the importance of *nos*-dependent transcriptional quiescence, *nos* PGCs can be partially rescued by mutations in one of the *nos* target genes *Sex-lethal* (Deshpande *et al.* 1999).

As for mechanisms, nothing is known about how *gcl* blocks transcription, whereas *nos* is thought to act by repressing the translation of an unknown general transcription factor. For *pgc*, the mechanism is well understood (Hanyu-Nakamura *et al.* 2008). Pgc protein interacts with transcription elongation factor b (P-TEFb), which is responsible for phosphorylating Ser2 residues in the CTD heptad repeat. The association of Pgc with P-TEFb prevents P-TEFb from being recruited to sites of paused polymerase. Consistent with this biochemical mechanism, PGCs in blastoderm-stage *pgc* mutants have high levels of CTD-PSer2. Moreover, targeting transcriptional elongation seems to be a conserved mechanism for imposing transcriptional quiescence, as the *C. elegans* PIE-1 protein is also thought to arrest transcription by inhibiting P-TEFb (Seydoux *et al.* 1996; Batchelder *et al.* 1999; Nakamura and Seydoux 2008).

Although it has been suggested that Pgc and its target P-TEFb are the central players in establishing transcriptional quiescence in newly formed PGCs (Cinalli *et al.* 2008; Nakamura and Seydoux 2008), it is striking that loss of *pgc* does not fully disrupt the specification of PGC fate or their eventual transition into functional GSCs. This would suggest either that the establishment and/or maintenance of transcriptional quiescence is not a necessary step in PGC development in Drosophila or that pgc has an important, but not absolutely essential, role in this process. For these reasons, we have re-examined the functioning of *pgc* in germline development and explored its relationship to the PGC/GSC-determinant *nos*.

## Materials and Methods

### Fly stocks and culture

Flies were grown at room temperature (22°) on standard medium. The following stocks obtained from Bloomington Stock Center were used for analysis: *nanos-Gal4:VP16*, *twist-Gal4*, *nos^BN^*, *nos^RC^*. Also used were two different extensively characterized loss-of-function alleles of *pgc*: *pgc^[EY09338]^* and *pgc^[EY07794]^* (Martinho *et al.* 2004). The loss of Nos protein in pgc PGCs was confirmed using AS-26, antisense pgc transgene (Nakamura *et al.* 1996). Nos-tubulin 3′ UTR transgenes were a kind gift from Liz Gavis, Princeton University. Typically, virgin females homozygous for the *Gal4* transgene were mated with males carrying the *UAS* transgene (Brand and Perrimon 1993). Embryos from these crosses were fixed and stained for subsequent analysis.

### Immunohistochemistry

The stainings were performed essentially as described in Deshpande *et al.* (1995). For immunofluorescence-based stainings, to begin with, the optimal settings were determined using wild-type control embryos. After the initial optimization, the alterations were kept to a minimum during the experiment. Multiple embryos and/or PGCs were imaged to assess the relevant differences in concentrations. Also, to minimize the variation, wherever possible, alterations in the levels were assessed using the immediately adjacent PGCs belonging to the same embryo. Vasa antibody was either a rat or rabbit polyclonal used at 1:500 dilution. Anti-Nanos is a rabbit polyclonal antibody used at 1:500 dilution (Hanyu-Nakamura *et al.* 2008). Alpha-Spectrin and Hu-li tai shao mouse monoclonal antibodies were used at 1:4 dilution (from Developmental Studies Hybridoma Bank).

## Results and Discussion

### Subset of *pgc* pole cells displays loss of Nos protein

**Figure 1:**
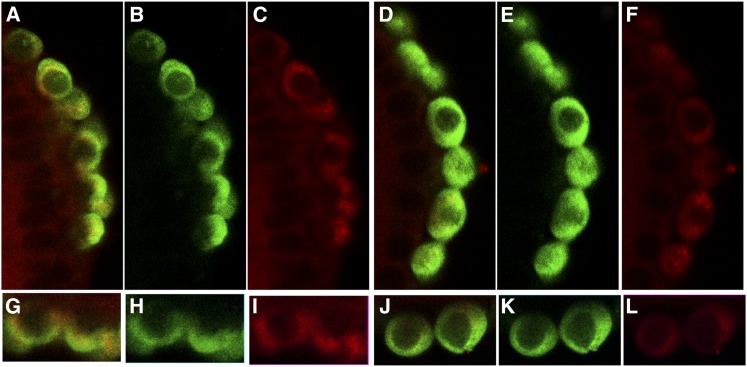
Nos protein levels are diminished in *pgc*- PGCs. Wild-type (panels A–C and G–I) and *pgc* (panels D–F and J–L) blastoderm-stage embryos were probed with Vasa (green) and Nos (red) antibodies. Panels A, D, G, and J show the merged images; panels B, E, H, and K show Vasa alone; and panels C, F, I, and L show Nos alone. Although levels of Nos are reduced, sometimes substantially in a subset of *pgc* PGCs, there is no apparent alteration in the levels of Vasa protein at the blastoderm stage. The difference and heterogeneity in Nos level is apparent in panels G–L, which show magnified images of two pole cells each. Similar results were obtained in three independent trials. The numbers in the text are from one of these experiments.

**Figure 2:**
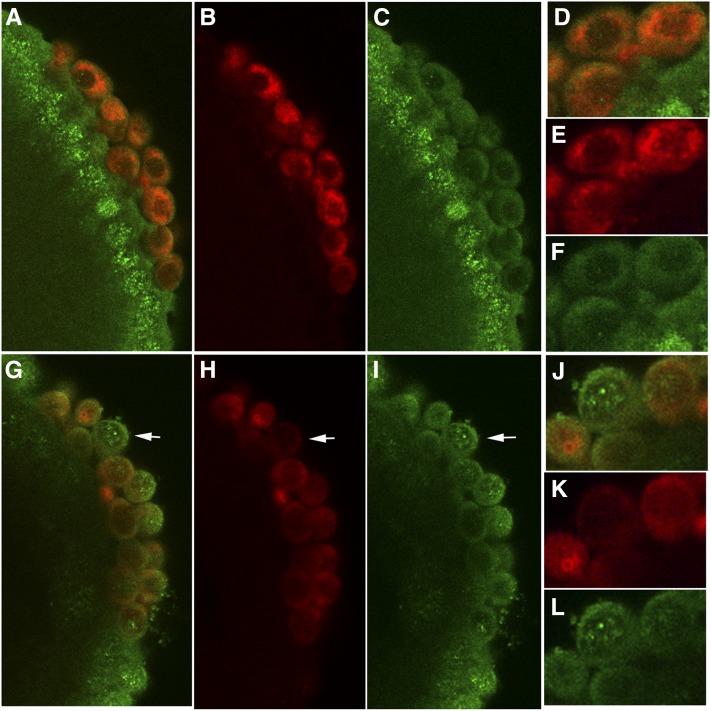
Loss of Nanos protein is in *pgc*^-^ PGCs is correlated with increased CTD PSer5. Stage 5 embryos from either wild-type mothers (A–C) or *pgc* mothers (D–F) were coimmunostained with Nos (red) and Pser5 (green) antibodies. Many *pgc*^-^ PGCs have reduced levels and/or uneven distribution of Nos (compare panels E and K). PGCs with reduced Nos have elevated Pser5 (panels F and L). One of the two different *pgc* pole cells displays higher levels of Nos protein and corresponding decreases in Pser5 levels. By contrast, wild-type PGCs show only low levels of signal compared with surrounding somatic nuclei (panels D–F). Similar results were obtained in two independent experiments. The numbers in the text are from one of the experiments. Arrows point to *pgc* PGC with reduced Nos and elevated PSer5.

Although Nakamura *et al.* (1996) have reported that *nos* mRNA levels in PGCs are reduced when *pgc* activity is compromised by an antisense *pgc* transgene, it has been argued that this diminution of *nos* mRNA is too slight and takes place too late to be relevant for *pgc* function (Martinho *et al.* 2004). However, Nos protein accumulation in PGCs compromised for *pgc* was not examined, and it seemed possible that the loss of *pgc* activity might have a greater effect on protein expression than it does on *nos* message levels. For this reason, we examined Nos accumulation in syncytial and cellular blastoderm stage in both antisense embryos (see supporting information, Figure S1) and embryos produced by two different *pgc* mutant alleles (Figure 1). In all three cases, we found that PGCs compromised for *pgc* activity exhibit an unusual, heterogeneous pattern of Nos accumulation. As illustrated for a stage 4 *pgc* embryo in Figure 1, the pattern of Nos protein accumulation in *pgc* PGCs is quite different from that in wild-type. In wild-type embryos, Nos protein accumulates to essentially the same level in most all PGCs (Figure 1; see also Figure 2 and Figure S1). By contrast, Nos levels are quite variable in *pgc* PGCs (Figure 1; see also Figure 2 and Figure S1). Some PGCs have near wild-type levels of Nos, others have intermediate amounts, and still others have little or no Nos. This highly heterogeneous pattern of Nos accumulation is evident soon after PGC formation and continues beyond the cellular blastoderm stage. Overall, over half of the *pgc* PGCs (58%; 75 out of 126 PGCs) in syncytial and cellular blastoderm-stage embryos have clearly reduced levels of Nos protein, whereas in wild-type, Nos levels are reduced in less than 10% (7%; 6 out of 91 PGCs) of the PGCs. From these findings, we conclude that the previously reported diminution of *nos* mRNA impacts Nos accumulation, but significantly, it does so only in a subset of the PGCs.

To determine whether the effects on Nos are specific, we examined the accumulation of another germline-specific translation factor, Vasa. As shown in Figure 1, Vasa levels in blastoderm-stage *pgc* PGCs are unaffected and resemble wild-type PGCs. However, later in development, Vasa levels are often substantially reduced in *pgc* PGCs that are undergoing apoptosis.

### Initiation CTD phosphorylation PSer5 is upregulated in a subset of *pgc* PGCs

An intriguing question is whether the reduction in Nos protein in a subset of the *pgc* PGCs has any impact on the development of these cells. If it does, one would expect to find that some *pgc* PGCs exhibit characteristic *nos*-like phenotypes, while others do not. Although both *pgc* and *nos* are known to play important roles in establishing transcriptional quiescence, they interfere with polymerase activity at different steps. Elegant studies by Hanyu-Nakamura *et al.* (2008) have shown that Pgc imposes transcriptional quiescence by specifically inhibiting the P-TEFb–dependent transcriptional elongation CTD phosphorylation, PSer2. By contrast, *nos* appears to downregulate transcription in PGCs at an earlier step in the transcription cycle, as the levels of both the initiation CTD phosphorylation, PSer5, and the elongation CTD phosphorylation, PSer2, are elevated in *nos* mutant PGCs (Deshpande *et al.* 2005).

If the loss of Nos in *pgc* PGCs affects their specification, then we would expect to find that PSer5 is upregulated in cells that have reduced amounts of Nos. Before testing this possibility, we first confirmed previous reports that PSer2 is elevated in all *pgc* PGCs (data not shown). We next examined PSer5 in wild-type, *nos*, and *pgc* PGCs. As shown for one of the *pgc* alleles in Figure 2, although PSer5 is clearly present in wild-type PGCs, the level of this CTD phosphorylation is substantially reduced compared with nearby somatic nuclei (n = 70). Similar results were obtained for the other *pgc* allele and for the *pgc* antisense. By contrast, in *nos* embryos, essentially all PGCs have levels of PSer5 approaching that in the surrounding somatic nuclei (see Figure S2). As was observed for Nos protein, there is a quite heterogeneous pattern of PSer5 in *pgc* PGCs. As shown in Figure 2, some *pgc* PGCs resemble wild-type and have little PSer5. However, a subset of the PGC nuclei have levels of PSer5 approaching that found in somatic nuclei. A careful analysis of multiple *pgc* embryos indicates that the extent of upregulation of PSer5 is variable and that elevated levels of this CTD phosphorylation are seen unambiguously in only about 50% of stage 4 or 5 PGCs (n = 100). In the remaining PGCs, there was either no increase or only a marginal increase.

We next asked whether the *pgc* PGCs with elevated nuclear PSer5 are the ones with reduced amounts of Nos. To test this possibility, we examined PGCs in stage 5 (cellular blastoderm embryos) coimmunostained with Pser5 and Nos antibodies. Figure 2 shows that the subset of PGCs with reduced Nos corresponds closely to the subset with elevated PSer5. In this experiment, we found that 61% (32 out of 53) of the *pgc* pole cells had reduced amounts of Nos. Of the pole cells with reduced Nos, PSer5 was elevated in nearly 90% (27 out of 30). Significantly, none of the PGCs with normal levels of Nos had elevated PSer5. We next examined stage 4 embryos to test whether Nos protein levels are diminished in *pgc* PGCs in stage 4 embryos. As can be seen from embryo shown in Figure S3, Nos protein levels are non-uniform in *pgc* PGCs even at this earlier stage. Moreover, in the PGCs with reduced Nos, there is concomitant increase in PSer5 levels.

### Does loss of Nos in *pgc* PGCs have other phenotypic consequences?

The results described above argue that the CTD initiation phosphorylation is upregulated in a subset of *pgc* mutant PGCs because they lack sufficient Nos. If this conclusion were correct, then we would expect an approximately similar fraction of the *pgc* PGCs to exhibit other phenotypes characteristic of *nos* PGCs, such as migration defects, upregulation of Cyclin B, and premature division, cell death, and a failure to initiate the transition from PGC to GSC identity. Consistent with these expectations, Nakamura *et al.* (1996) reported that a subset of the PGCs in progeny of antisense *pgc* mothers exhibited migration defects and died. We confirmed the migration defects in progeny *pgc* mutant mothers. We also found that the coalesced gonads of stage 14 mutant embryos had fewer PGCs. Whereas wild-type have 9.5 PGCs/gonad (n = 100), *pgc* embryos have on average 3.5 PGCs/gonad (n = 75). To address this issue further, we determined whether *pgc* PGCs exhibit other *nos*-like phenotypes.

**Figure 3:**
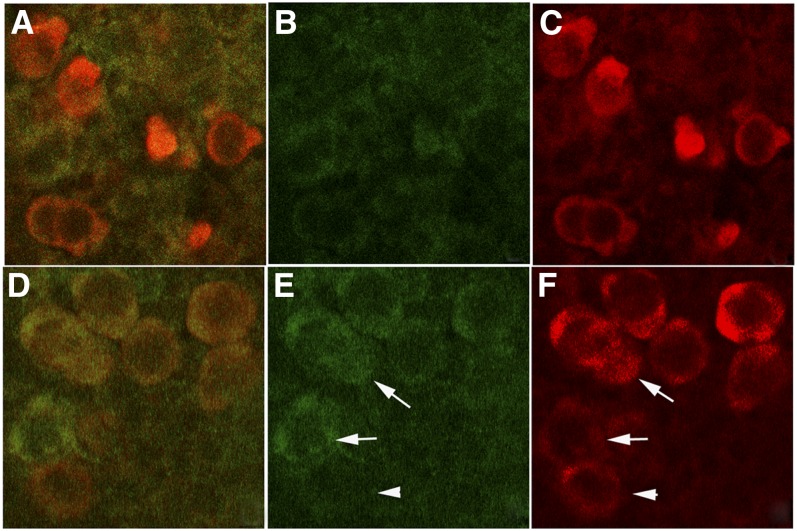
Cyclin B is prematurely expressed in *pgc* PGCs. Wild-type (A, B) and *pgc* (C, D) embryos were probed with Vasa (red) and Cyclin B (green) antibodies. Panels A and C show the merged image; panels B and E show Cyclin B protein alone; and panels C and F show Vasa protein only. Shown here are stage 10 wild-type and *pgc* embryos. Whereas Cyclin B is infrequently observed in wild-type stage 10 PGCs (5%; 1 out of 19), the majority of the *pgc* PGCs express detectable levels of Cyclin B at this stage of development (68%; 17 out of 25). Three independent experiments yielded similar results. Also, note that inappropriate expression of Cyclin B can be detected in *pgc* PGCs from younger blastoderm-stage embryos; however, Cyclin B–positive PGCs are much less frequent at earlier stages. Arrows in panels E and F indicated PGCs with elevated levels of Cyclin B, while the arrowhead indicates a PGC with little or no Cyclin B.

Premature cell division in *nos* PGCs is due to the inappropriate expression of the mitotic cyclin Cyclin B (Asaoka *et al.* 1999). In wild-type PGCs, Nos together with Pumilio repress the translation of *cyclinB* mRNA and PGCs arrest the mitotic cycle in G2. To determine whether loss of Nos in *pgc* PGCs results in the premature expression of Cyclin B protein, we probed wild-type and *pgc* embryos with Cyclin B antibodies. As shown in Figure 3, a substantial fraction of *pgc* PGCs in stage 10 embryos (70%; >100 PGCs) have Cyclin B, whereas Cyclin B is infrequently detected in PGCs of wild-type embryos (5%; >100 PGCs) of the same stage. In fact, in wild-type embryos, Cyclin B is not upregulated until much later in development after gonad coalescence (Asaoka *et al.* 1999).

**Figure 4:**
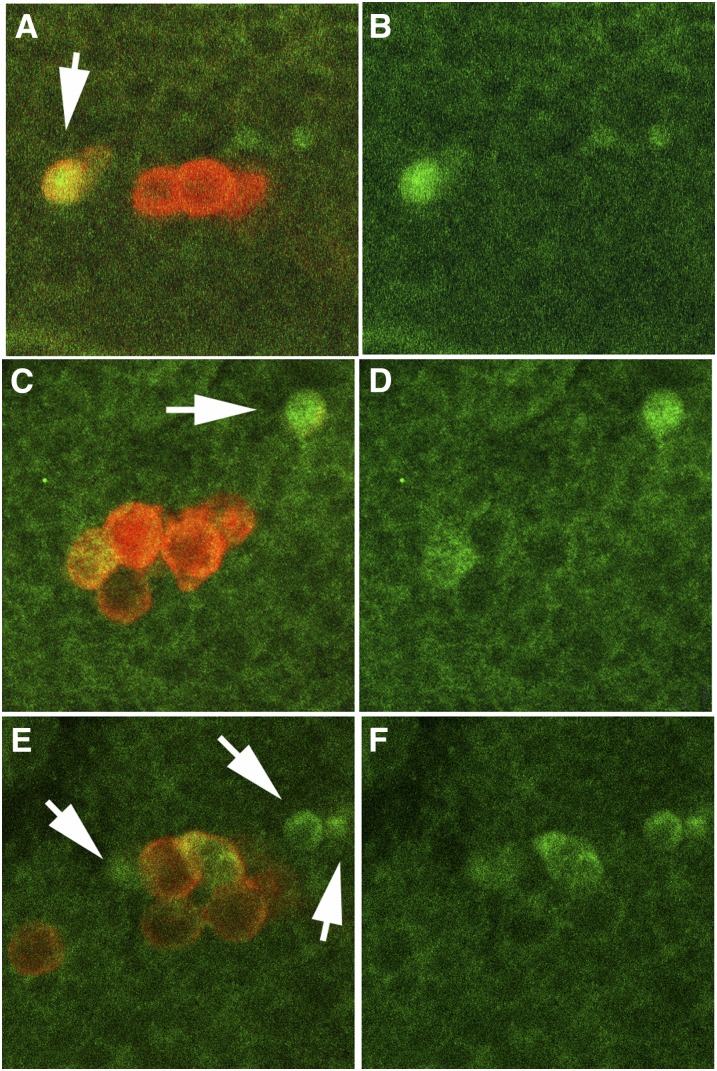
*pgc*^-^ pole cells undergo apoptosis. Stages 13–15 wild-type (not shown) and *pgc*^-^ embryos were probed with activated Caspase3 (green) and Vasa (red) antibodies. Activated caspase3 is only present in cells undergoing apoptosis. Panels A, C, and G show both Vasa and activated Caspase3; panels B, D, and F show only activated Caspase3. Arrows indicate Vasa-positive cells with activated Caspase3. In some cases, little Vasa remains. No activated Caspase3 was detected in PGCs of similarly staged wild-type embryos (n = 20 embryos; >100 PGCs). For this reason, the control is not shown here. The experiment was repeated twice; numbers in the text represent a single trial.

Cell death in *nos* PGCs is due to activation of the *head involution defective* apoptosis pathway Sato *et al.* (2007) and Maezawa *et al.* (2009) have shown that over 20% of the PGCs in stages 12–16 *nos* embryos express the cell death marker cleaved Caspase3. We used antibodies specific for cleaved Caspase3 to test whether this pathway is also activated in *pgc* PGCs. As Figure 4 shows, all *pgc* embryos examined had at least one PGC that was positive for cleaved Caspase 3. Many of the activated Caspase3-positive PGCs also had greatly reduced Vasa (arrows). As Vasa differed from Nos in that it was not lost in cellular blastoderm *pgc* PGCs, we suspect that this is a consequence of cell death rather than some more direct function of *pgc* in sustaining Vasa protein levels.

The transition from PGC to GSC identity begins with assembly of a germ cell–specific organelle called the spectrosome (Lin *et al.* 1994; Lin and Spradling, 1995). Spectrosome-like structures can first be detected at stage 11, just after the PGCs exit the midgut and start migrating through the mesoderm. Between stages 11 and 15 of embryogenesis as PGCs complete their migration and coalesce into the embryonic gonad, the spectrosome enlarges progressively. By stage 15, it is spherical in shape and closely resembles the structure found in adult GSCs.

**Figure 5:**
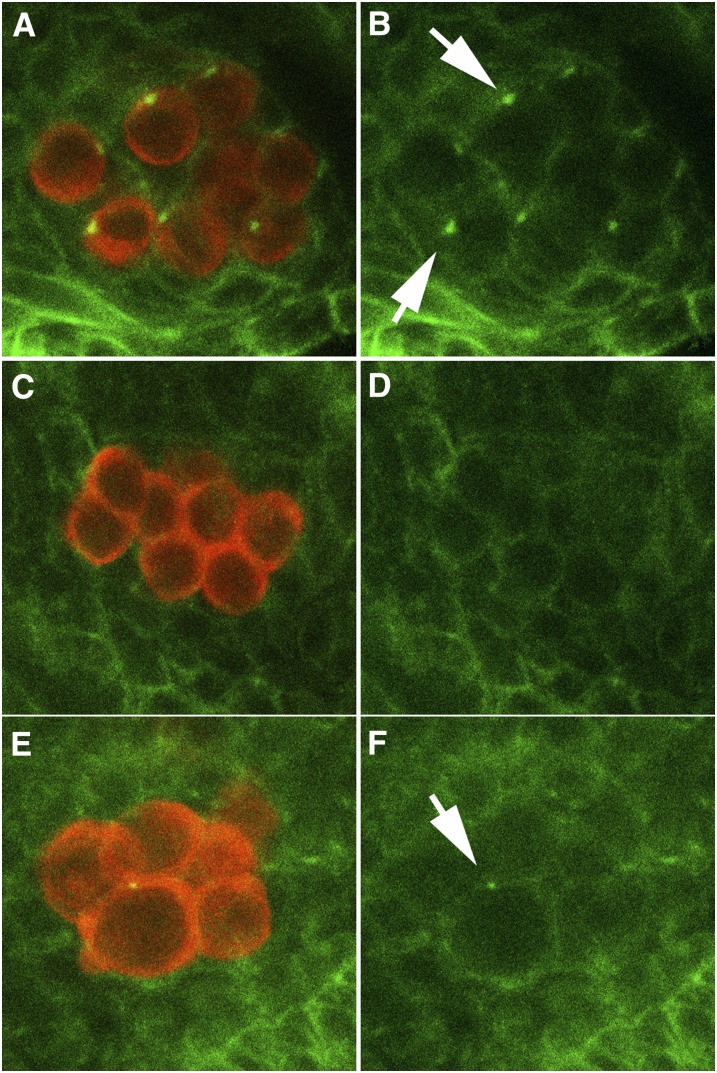
Defective spectrosomes in *pgc*^-^ PGCS. Wild-type and *pgc*^-^ embryos were probed with alpha-Spectrin (green) and Vasa (red) antibodies. Panels A, C, and E show both the signals, whereas panels B, D, and F show only the Spectrin-specific signal. Wild-type germ cells in coalesced embryonic gonads have characteristic large, spherical, GSC-like spectrosomes (panel B), whereas *pgc*^-^ germ cells either lack spectrosomes altogether (panel D) or have incompletely formed spectrosomes (panel F). Arrows indicate GSC-like spectrosomes in wild-type and *pgc*^-^ PGCs.

Wawersik and Van Doren (2005) have shown that *nos* is required to initiate and maintain the assembly of spectrosomes in migrating PGCs of stages 11 and 12 embryos, and in *nos* mutants, spectrosomes are not detected in more than 90% of PGCs. To test whether spectrosome assembly is also disrupted in the progeny of *pgc* mothers, we probed wild-type and *pgc* embryos with spectrin antibodies. We found that newly formed spectrosomes in PGCs of stages 11 and 12 *pgc* embryos are typically smaller than the spectrosomes in wild-type embryos of the same age (not shown). As shown in Figure 5, abnormalities in spectrosome assembly were even more apparent after gonad coalescence. In wild-type stage 14 gonads (Figure 5A), all PGCs have a large, brightly stained spherical spectrosome (Figure 5, arrows). In contrast, in *pgc* mutants spectrosomes are missing altogether (Figure 5, B and C) or not fully developed (Figure 5F, arrow). About 50% (n = 100) of the surviving PGCs lack spectrosomes altogether; however, as with the other *nos*-like *pgc* phenotypes, we observe PGCs that have “wild-type” spectrosomes (not shown). (Note that it was not possible to draw the correlation between lack of Nos and missing spectrosomes, activated Caspase 3, or premature Cyclin B expression, as we were unable to label PGCs after they were carried inside the gut during gastrulation with the Nos antibody).

### Is *nos* a critical *pgc* target?

The results described above indicate that in addition to inappropriately upregulating CTD initiation phosphorylation, several *nos* phenotypes evident in PGCs of older embryos are recapitulated in *pgc* mutants. One hypothesis to explain this connection is that a critical function of *pgc* in PGC specification and development is to ensure proper Nos accumulation. In this scenario, PGCs in *pgc* embryos that have reduced Nos would not be properly specified, have defects in migration, and undergo apoptosis. Moreover, all of these phenotypes would be the consequence of failing to maintain sufficient amounts of Nos. In contrast, the *pgc* PGCs that are able to coalesce properly with the somatic gonad and develop into functional GSCs would be limited (at the minimum) to those that maintain sufficient levels of Nos. If this hypothesis were correct, it should be possible to rescue *pgc* PGCs by providing Nos protein.

**Figure 6:**
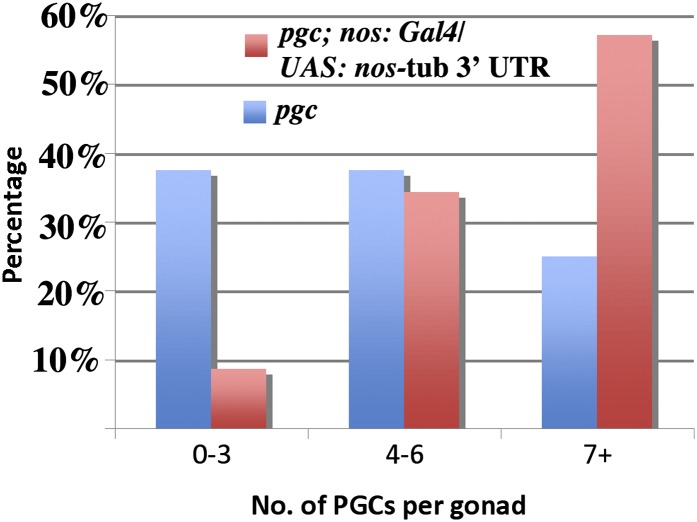
Germ-cell loss in *pgc* mutants is partially rescued by ectopic Nos. Blue and red bars correspond to PGCs/gonad in *pgc* and *pgc*; *nos: Gal4/*UAS *nos: tubulin* 3′ UTR embryos, respectively. The embryonic gonads were classified based on total number of surviving PGCs at stage 13 and beyond. This experiment was done twice. Both experiments gave similar distributions and are tabulated together in the text.

For this purpose, we used the Gal4/UAS system. Because the *nos* 3′UTR contains elements that control localization, translation, and stability, we used an UAS transgene carrying the *nos* coding sequence fused to the *tubulin* 3′ UTR (Bergsten and Gavis 1999). To drive expression specifically in the germline, we used a *nos*:Gal4 transgene. As the *nos* promoter is not activated in PGCs until after they exit the midgut and begin migrating toward the somatic gonad, we anticipated that if supplying Nos rescues the *pgc* PGCs, rescue should be at best incomplete because of this delay. Figure 6 shows that the effects of *pgc* on the migration and viability of PGCs can be partially rescued by providing Nos. While embryonic gonads of *pgc* mutants have on average 3.5 PGCs (n = 32), *pgc* mutants carrying the *nos*: Gal4/UAS: *nos*-Tublin 3′ UTR transgene combination had on average 7 (n = 35; *P* value = 1.918 × 10^−5^). The effects of supplying Nos can also be seen in the upward shift in the number of PGCs in embryonic gonads (*e.g.* nearly 40% of the *pgc* embryos have 3 or fewer PGCs/embryonic gonad, whereas about 90% of the rescued embryos had 4 PGCs or more).

The finding that PGCs in *pgc* mutant embryos can be rescued by supplying Nos indicates that one critical *pgc* function is to ensure that there are sufficient amounts of Nos. This conclusion fits with the *nos*-like phenotypes evident in a subset of *pgc* mutant PGCs. At the blastoderm stage, the subset of PGCs that lose Nos also fail to prevent upregulation of the CTD initiation phosphorylation PSer5, which is the target for *nos*-dependent transcriptional quiescence. Although it wasn’t possible to draw a similar connection in the older *pgc* embryos, we nevertheless found that a substantial fraction of the PGCs exhibit phenotypes characteristic of *nos* mutations (migration defects, premature Cyclin B expression, failure to initiate spectrosome assembly, and activation of apoptosis). Because rescue in our experiment was incomplete, it could be argued that *pgc* has functions that are important for the specification and development of PGCs in addition to maintaining high levels of Nos. On the other hand, not all *pgc* PGCs lose Nos (at the blastoderm stage). Consequently, it remains possible that all that is needed for *pgc* PGCs to form fully functional GSCs is that they retain sufficient levels of Nos. If this were true, then the only important (but not absolutely essential) function of *pgc* would be to ensure that the levels of Nos in PGCs remain high enough so that it can properly specify PGC/GSC fate.

While our findings argue that maintaining full *nos* activity is a critical function of *pgc*, it is not clear which of the known *nos* regulatory targets is primarily responsible for the detrimental effects on PGC development. Previous studies indicate that an important function for *nos* in PGC development is to maintain transcriptional quiescence (Deshpande *et al.* 1999). Moreover, as is observed in *nos* mutant PGCs, the initiation CTD phosphorylation PSer5 is elevated in *pgc* PGCs that have reduced levels of Nos protein but not in PGCs that have wild-type levels of Nos. Although this correlation would seem to point to *nos*-dependent transcriptional quiescence, misexpression of two known *nos* transcriptional targets, *Sxl* and *even-skipped*, was not detected in *pgc* PGCs (Deshpande *et al.* 2004; Martinho *et al.* 2004). Thus, it is possible that the detrimental effects of reduced Nos in *pgc* PGCs arises from a failure to regulate one of the other *nos* targets, for example Cyclin B mRNA translation. On the other hand, while Nos protein is reduced in amount in a subset of blastoderm-stage *pgc* PGCs, it is not completely eliminated at this point in development, and further reductions might occur as embryogenesis proceeds. In this case, it is possible that some of the *nos* transcriptional targets become activated a bit later in development. An additional caveat is that the methods used to assay *Sxl* and *even-skipped* expression were not the most sensitive, and a low level of expression of these genes in a subset of the blastoderm *pgc* PGCs could have been missed.

The idea that *pgc* might play a subordinate role to *nos* in germline development and that its primary function is to ensure that Nos can specify PGC/GSC fate would dovetail nicely with recent studies of Lai *et al.* (2012) on the development of the primordial germline in the vertebrate *Xenopus*. As has been previously reported for the functioning of *nos* during germline development in the *Drosophila* embryo, Lai and coauthors found that *Xenopus nos* is required for transcriptional quiescence, for germ cell survival and migration to the somatic gonad, and for the process of germ cell specification. In this context, it is also notable that the *nos* gene is widely conserved across the animal kingdom, as is its function in the process of PGC/GSC specification [for example, see Suziki *et al.* (2007) and Tsuda *et al.* (2003)]. By contrast, *nos* function in abdominal segmentation in *Drosophila* appears to be a specialized adaption that is likely restricted to insects. Similarly, the *pgc* gene is not well conserved. These differences would be consistent with the speculation that a *pgc*-like activity evolved in flies (and presumably other insects) to ensure that the functioning of *nos* in germline development is not compromised by the quite different requirements for its activity and its regulation in the development of the posterior soma.

## Supplementary Material

Supporting Information
