# Supplementary material for: Maintaining Sufficient Nanos Is a Critical Function for Polar Granule Component in the Specification of Primordial Germ Cells
Source: G3 (Bethesda). 2012 Nov 1;2(11):1397–403. doi: 10.1534/g3.112.004192 (PMC3484670; doi:10.1534/g3.112.004192)
Supplement: Supporting Information [file supp_2_11_1397__index.html]

Supporting Information 

# Maintaining Sufficient Nanos Is a Critical Function for *Polar Granule Component* in the Specification of Primordial Germ Cells

## Supporting Information for Deshpande *et al.*, 2012

**Files in this Data Supplement:**

- Supporting Information - Figures S1-S3 (PDF, 4 MB)
- Figure S1 - Nos protein levels are reduced in anti-sense pgc PGCs (PDF, 820 KB)
- Figure S2 - Phosphorylation of Polymerase II CTD PSer5 is elevated in all the *nos* PGCs (PDF, 2.8 MB)
- Figure S3 - Correlation between the loss of Nanos protein with increased CTD PSer5 is observed in stage 4 *pgc*-PGCs (PDF, 465 KB)
