# Supplementary material for: Maintaining Sufficient Nanos Is a Critical Function for Polar Granule Component in the Specification of Primordial Germ Cells
Source: G3 (Bethesda). 2012 Nov 1;2(11):1397–403. doi: 10.1534/g3.112.004192 (PMC3484670; doi:10.1534/g3.112.004192)
Supplement: Supporting Information [file supp_2.11.1397_004192SI.pdf]

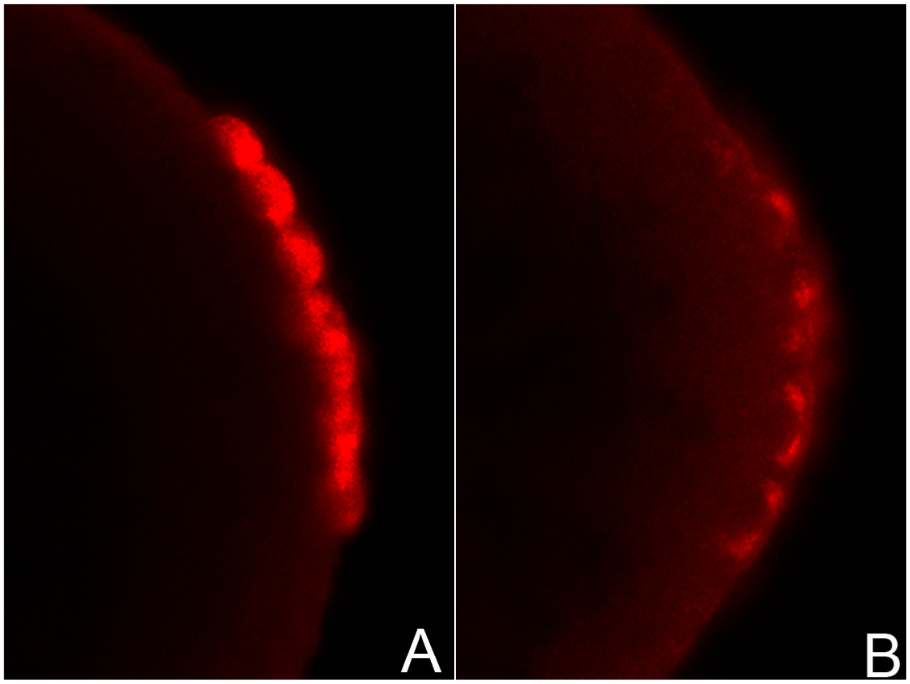

Figure S1 Nos protein levels are reduced in anti-sense *pgc* PGCs. Wild type (A) and anti-sense *pgc* (B) blastoderm stage embryos were probed with Nos (red) antibodies. Levels of Nos were found to be reduced in more than 60% (n=35) of PGCs.

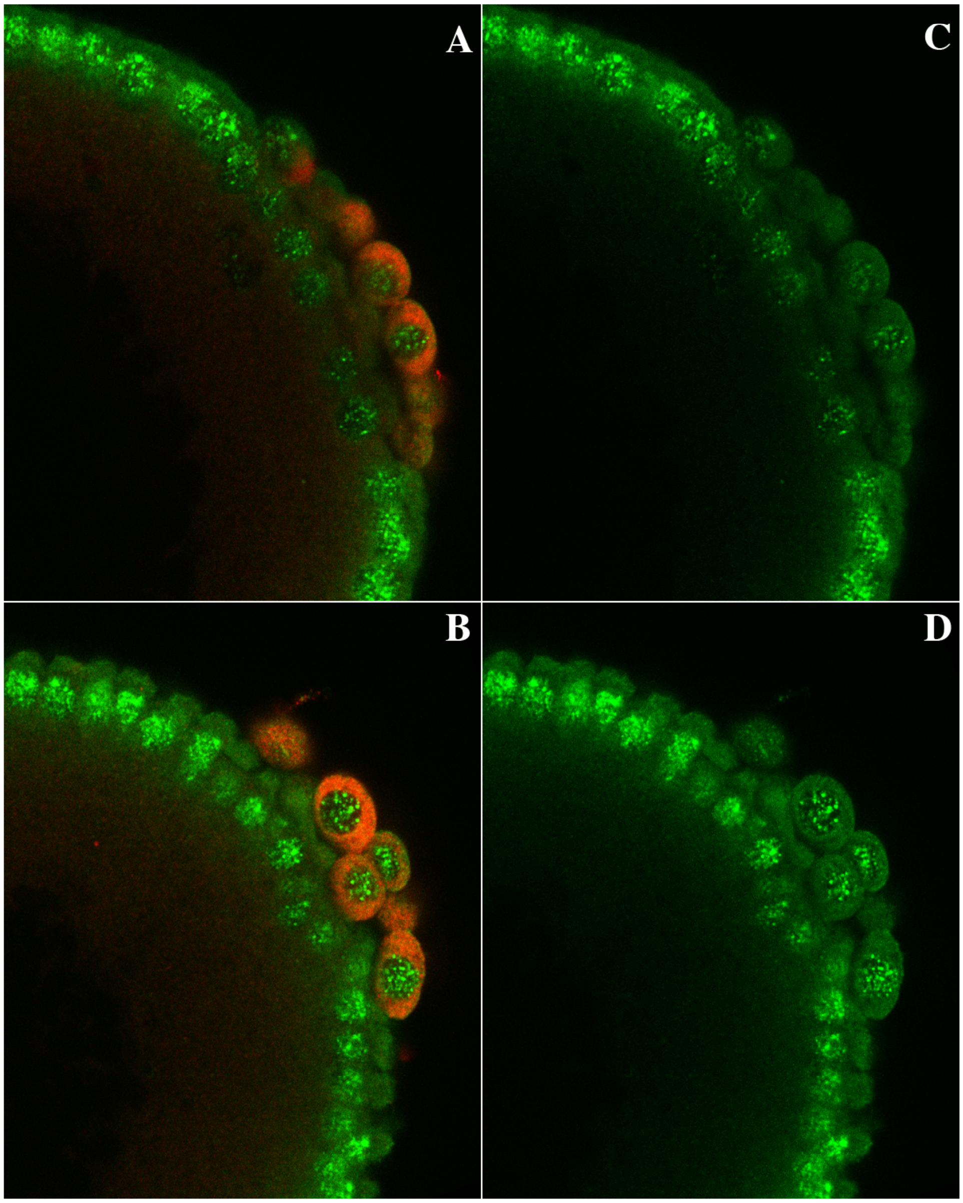

Figure S2 Phosphorylation of Polymerase II CTD PSer5 is elevated in all the *nos* PGCs. Wild-type (A) and *nos* (B) embryos probed with CTD PSer5-specific antibody (green) and Vasa (red) antibody. A low level pf PSer5 is typically detected in wild type PGCs. In *nos* mutants, the CTD initiation phosphorylation is elevated in all PGCs.

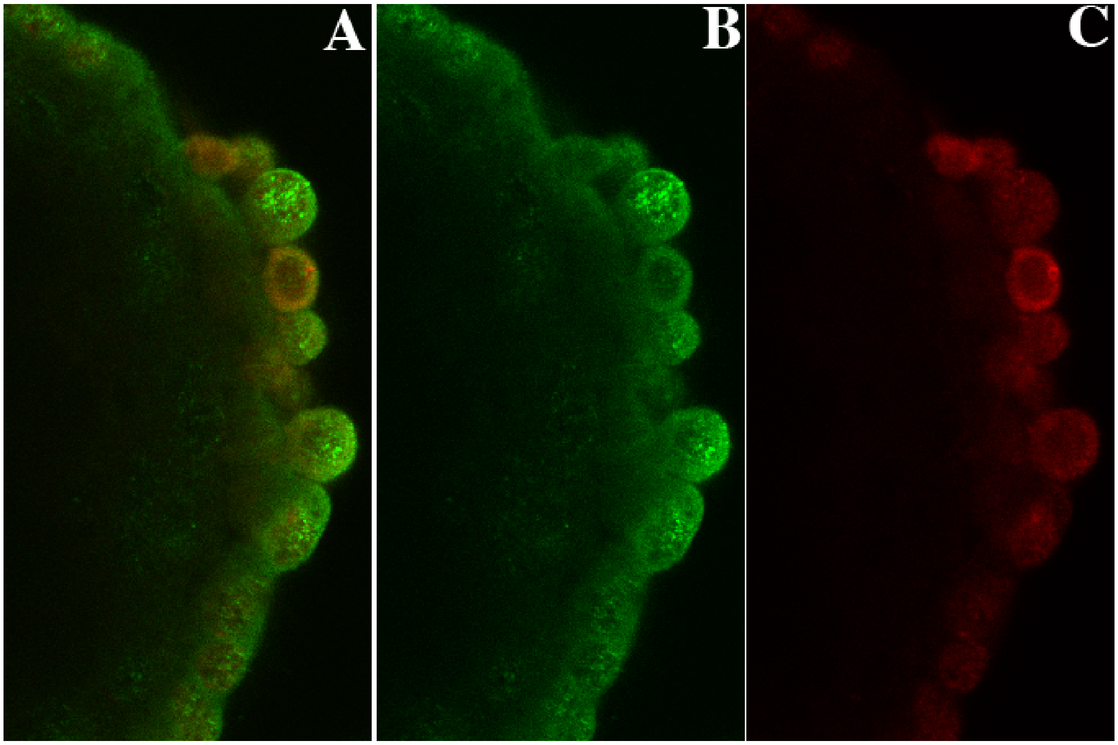

Figure S3 Correlation between the loss of Nanos protein with increased CTD PSer5 is observed in stage 4 *pgc*$^{-}$PGCs. Progeny of wild type (not shown: see Fig.2) or *pgc* mothers were probed with Nos (red) and Pser5 antibody. Many *pgc*$^{-}$ PGCs have reduced levels and/or uneven distribution of Nos. PGCs with reduced Nos have elevated Pser5.
