# Supplementary material for: Maintaining Sufficient Nanos Is a Critical Function for Polar Granule Component in the Specification of Primordial Germ Cells
Source: G3 (Bethesda). 2012 Nov 1;2(11):1397–403. doi: 10.1534/g3.112.004192 (PMC3484670; doi:10.1534/g3.112.004192)
Supplement: Supporting Information [file supp_2.11.1397_FigureS1.pdf]

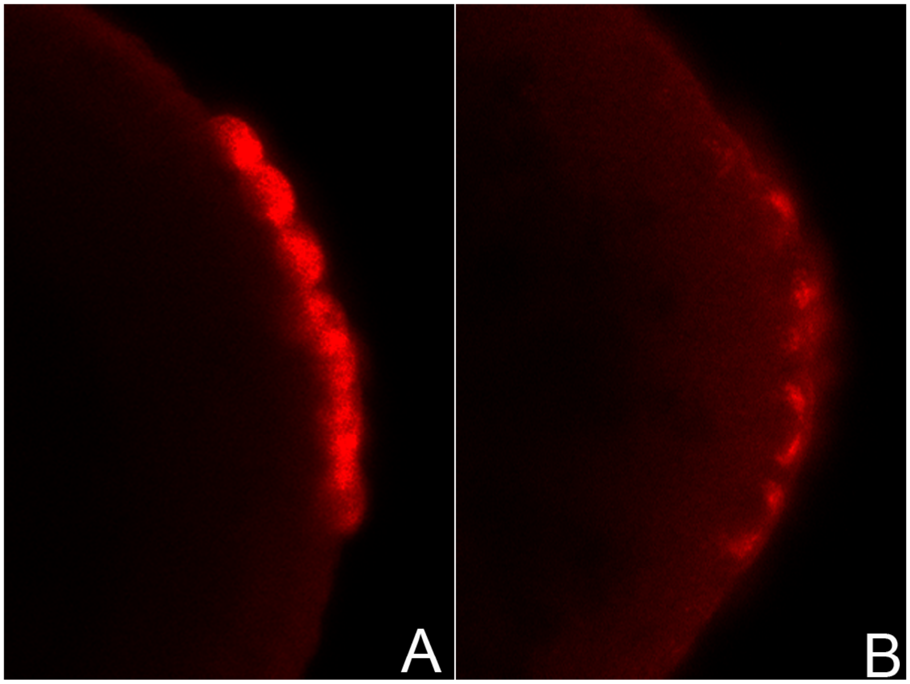

Figure S1 Nos protein levels are reduced in anti-sense *pgc* PGCs. Wild type (A) and anti-sense *pgc* (B) blastoderm stage embryos were probed with Nos (red) antibodies. Levels of Nos were found to be reduced in more than 60% (n=35) of PGCs.
