# Supplementary material for: Maintaining Sufficient Nanos Is a Critical Function for Polar Granule Component in the Specification of Primordial Germ Cells
Source: G3 (Bethesda). 2012 Nov 1;2(11):1397–403. doi: 10.1534/g3.112.004192 (PMC3484670; doi:10.1534/g3.112.004192)
Supplement: Supporting Information [file supp_2.11.1397_FigureS2.pdf]

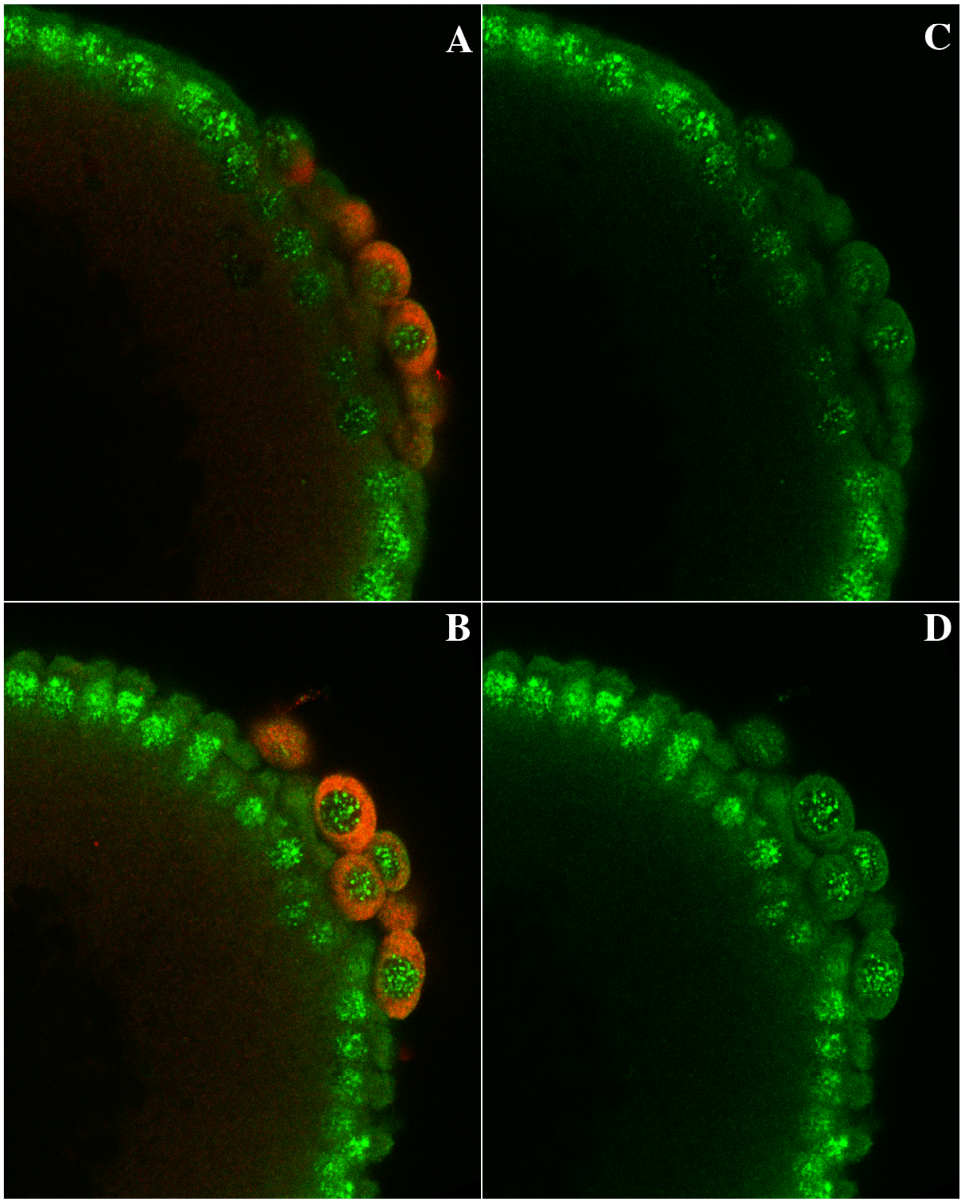

Figure S2 Phosphorylation of Polymerase II CTD PSer5 is elevated in all the *nos* PGCs. Wild-type (A) and *nos* (B) embryos probed with CTD PSer5-specific antibody (green) and Vasa (red) antibody. A low level pf PSer5 is typically detected in wild type PGCs. In *nos* mutants, the CTD initiation phosphorylation is elevated in all PGCs.
