# Supplementary material for: Maintaining Sufficient Nanos Is a Critical Function for Polar Granule Component in the Specification of Primordial Germ Cells
Source: G3 (Bethesda). 2012 Nov 1;2(11):1397–403. doi: 10.1534/g3.112.004192 (PMC3484670; doi:10.1534/g3.112.004192)
Supplement: Supporting Information [file supp_2.11.1397_FigureS3.pdf]

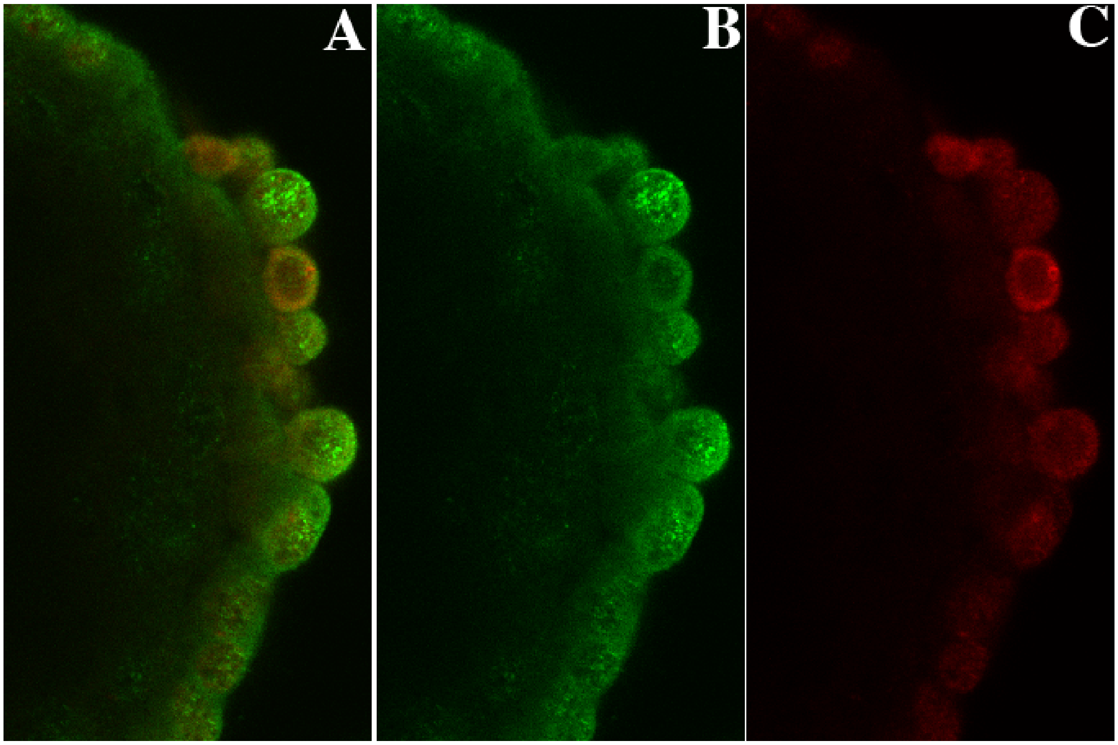

Figure S3 Correlation between the loss of Nanos protein with increased CTD PSer5 is observed in stage 4 *pgc*$^{-}$PGCs. Progeny of wild type (not shown: see Fig.2) or *pgc* mothers were probed with Nos (red) and Pser5 antibody. Many *pgc*$^{-}$ PGCs have reduced levels and/or uneven distribution of Nos. PGCs with reduced Nos have elevated Pser5.
